# Mycorrhizal lipochitinoligosaccharides (LCOs) depolarize root hairs of *Medicago truncatula*

**DOI:** 10.1371/journal.pone.0198126

**Published:** 2018-05-31

**Authors:** Anna-Lena Hürter, Sébastien Fort, Sylvain Cottaz, Rainer Hedrich, Dietmar Geiger, M. Rob G. Roelfsema

**Affiliations:** 1 Molecular Plant Physiology and Biophysics, Julius-von-Sachs Institute for Biosciences, Biocenter, Würzburg University, Würzburg, Germany; 2 University Grenoble Alpes, CNRS, CERMAV, Grenoble, France; University of Tasmania, AUSTRALIA

## Abstract

Arbuscular Mycorrhiza and Root Nodule Symbiosis are symbiotic interactions with a high benefit for plant growth and crop production. Thus, it is of great interest to understand the developmental process of these symbioses in detail. We analysed very early symbiotic responses of *Medicago truncatula* root hair cells, by stimulation with lipochitinoligosaccharides specific for the induction of nodules (Nod-LCOs), or the interaction with mycorrhiza (Myc-LCOs). Intracellular micro electrodes were used, in combination with Ca^2+^ sensitive reporter dyes, to study the relations between cytosolic Ca^2+^ signals and membrane potential changes. We found that sulfated Myc- as well as Nod-LCOs initiate a membrane depolarization, which depends on the chemical composition of these signaling molecules, as well as the genotype of the plants that were studied. A successive application of sulfated Myc-LCOs and Nod-LCOs resulted only in a single transient depolarization, indicating that Myc-LCOs can repress plasma membrane responses to Nod-LCOs. In contrast to current models, the Nod-LCO-induced depolarization precedes changes in the cytosolic Ca^2+^ level of root hair cells. The Nod-LCO induced membrane depolarization thus is most likely independent of cytosolic Ca^2+^ signals and nuclear Ca^2+^ spiking.

## Introduction

Roots establish two symbiotic associations with microbes that both play an important role in plant nutrition. An ancient symbiosis with Arbuscular Mycorrhizal fungi (AM) is found in approximately 80% of land plants, which evolved roughly 450 million years ago [1, 2]. The Root Nodule Symbiosis (RNS) emerged only within the last 60 million years and is confined to the family of legume plants (*Fabaceae*) with the exception of *Parasponia* (*Cannabaceae*) [3, 4]. Both, AM and RNS are of great importance for plant nutrition, as AM fungi supply plants with various nutrients from the soil [5], whereas the rhizobium bacteria in the nodules provide fixed nitrogen sources [6, 7].

Early steps in the development of AM and RNS are very similar, despite of the large differences in their morphology and the nature of the symbiotic microbes. Both, AM fungi and nitrogen-fixing bacteria exudate diffusible symbiotic signals, consisting of a chitinoligosaccharide backbone (CO) that is composed of four, or five, *N*-acetylglucosamine residues (GlcNAc). An *N*-acyl group is attached to the non-reducing terminal sugar and gives rise to molecules defined as lipochitinoligosaccharides (LCOs) [8, 9]. LCOs involved in Nodulation (Nod) can have further substitutions on the glucosamine subunits, such as methylation, acetylation, glycosylation and sulfation, moreover the length and degree of saturation of the N-acyl residue can vary [10–12]. Less is known about the LCOs extruded by AM fungi, but *Glomus intraradices* exudates a mixture of sulfated, and non-sulfated, simple LCOs, whereas Palmitic acid (C16:0) and Oleic acid (C18:1) are the major N-acyl substitutions. Structural differences between Nod-and Myc-LCOs are limited. Nod-LCOs carry an *O*-acetyl group at position 6 of the non-reducing end, which is missing in Myc-LCOs [9].

During the development of AM and RNS symbiotic relations, LCOs are recognized by pairs of lysin motiv domain receptor like kinases (LysM-RLKs), located in the plasma membrane of the host plant cell. In *Medicago truncatula* Nod-LCOs are perceived by the LysM receptor kinase 3 (*Mt*LYK3) and Nod factor perception (*Mt*NFP) [13, 14], whereas the Nod factor receptors 1 (*Lj*NFR1) and *Lj*NFR5 were found in *Lotus japonicus* [15, 16]. *Mt*LYK3 forms a receptor complex with the leucin rich repeat receptor like kinase (LRR-RLK) *Mt*DMI2 (*Lj*SYMRK), which is also essential for Nod-LCO perception [17, 18]. These Nod-LCO receptors are homologous to the receptors for chitin fragments, which trigger Microbe Associated Molecular Patterns (MAMPs)-dependent pathogen defense responses [19, 20].

Because of the strong similarity between Nod- and Myc-LCOs, both stimuli may affect the plasma membrane potential in a similar manner. We therefore used intracellular electrodes to measure membrane potential changes of *Medicago truncatula* root hairs to Nod- and Myc-LCOs. This approach revealed that root hairs depolarize upon stimulation with sulfated Myc-LCOs, but not in response to non-sulfated Myc-LCOs. Sulfated Myc-LCOs repress responses to Nod-LCOs, which suggests that both stimuli share components of early steps in the signaling pathway. Interestingly, no correlation was observed between the depolarization and intracellular Ca^2+^ signals, which suggests that LCOs evoke a plasma membrane depolarization in a Ca^2+^-independent manner.

## Materials and methods

### Plant material and experimental conditions

Seeds of *Medicago truncatula* Gaertn cv. Jemalong A17 and cv. R108-1 were surface-sterilized with concentrated sulphuric acid for approximately 10 min, rinsed six times with sterile distilled water and then kept in 2% (v/v) sodium hypochloride for 2 min. Thereafter, seeds were washed with sterile distilled water six times and seed moisture expansion was allowed for 4 h before seeds were spread on 1% (w/v) agar in water (Agar Agar Kobe I, Roth, www.carlroth.com). Plates were sealed with parafilm (Parafilm, www.parafilm.com), inverted, wrapped with aluminum foil and vernalized in 4°C for 96 h [21, 22]. After vernalization, *M*. *truncatula* seeds were incubated for 24 h in the dark at room temperature. Germinated seeds were moisturized with sterile distilled water (pH 7.0) to allow the removal of seed coats. For microelectrode impalement of root hair cells, the *M*. *truncatula* seedlings were grown for 2 h in a climate chamber with a light intensity 84 μmol m^-2^ s^-1^ on agar plates with the following solution 0.1 mM CaCl_2_, 0.1 mM NaCl, 0.1 mM KCl and 5 mM MES/Tris, pH 7.0, supplemented with 1.5% (w/v) Plant Agar (Duchefa, www.duchefa-biochemie.com). Electrophysiological measurements were performed in a bath solution with 0.1 mM CaCl_2_, 0.1 mM NaCl, 0.1 mM CsCl and 5 mM MES/Tris, pH 7.0. Seedlings incubated with this bath solution for 1–2 h were fixed in an experimental chamber with double-sided adhesive tape. Roots were immobilized with small stripes of parafilm.

Three different Myc-LCOs, synthesized by a two steps chemo-biotechnological process [9, 23] were used. Myc-LCO-IV-C18:1 and Myc-LCO-IV-C18:1-S are both *N*-acylated by oleic acid (C18:1) and differ only in the presence or absence of a sulfate group (S) on one glucosamine subunit. Myc-LCO-IV-C16:0-S is also sulfated, but it is *N*-acylated by palmitic acid (C16:0). The three Myc LCOs, as well as the *Sinorrhizobium meliloti* Nod LCOs (NodSm-IV-C16:2-S), were dissolved in 50% (v/v) ethanol to obtain a 1 mM stock solution. For all measurements the Nod- and Myc-LCOs were diluted in bath solution to a concentration of 100 nM. LCOs were applied via a perfusion system based on gravitation.

### Experimental setup for impalement with microelectrodes and electrical configuration

The experimental chamber with a *M*. *truncatula* seedling was mounted in the focal plane of an upright microscope (Axioskop 2FS, Zeiss, http://www.zeiss.com). A water immersion objective (W Plan-Achroplan, 40x/0.8 W, Zeiss) was used to visualize the root hairs and to monitor the impalement of single root hairs with microelectrodes using a piezo-driven micromanipulator (MM3A, Kleindiek Nanotechnik, www.nanotechnik.com). The microelectrodes were pulled from borosilicate glass capillaries (inner diameter 0.58 mm, outer diameter 1.0 mm, with filament; Hilgenberg, www.hilgenberg-gmbh.com) using a horizontal laser puller (P2000; Sutter Instruments, www.sutter.com). The reference electrode consisted of a 300 mM KCl, 2% agarose salt bridge, connected to an Ag/AgCl half-cell and it was placed in the bath solution.

The microelectrodes were filled with 300 mM KCl for membrane potential recordings and connected via Ag/AgCl half-cells either to the head stage of an IPA-2 microelectrode amplifier (Applicable Electronics, www.applicableelectronics.com), or an HS180 headstage (HS180, BioLogic, http://www.bio-logic.info). The input resistance of the headstages was either >10^11^ (IPA-2) or 10^11^ Ω (HS180). Free running membrane potentials were recorded at 50 Hz, either using the WinEDR software [24, 25] (John Dempster, University of Strathclyde, spider.science.strath.ac.uk) with an NI USB 6259 interface (National Instruments, www.ni.com) or with the PULSE software (v. 8.74; Heka http://www.heka.com) using an LIH-1600 interface (HEKA). FURA2-dextran was current injected by applying a negative current of 4–10 nA for 1–2 min., through the electrode of which the tip was filled with 0.5 μl of 20 μM FURA2-dextran (Mw 10000, Thermo Fisher, www.thermofisher.com).

### Fluorescence microscopy

The fluorescent calcium indicator dye FURA2-dextran was excited by 200 ms UV flashlight from a VisiChrome high-speed polychromator system (Visitron Systems, Germany; www.visitron.de), at 345- and 395-nm wavelengths and a time interval of 5 s [26, 27]. The FURA2-Dextran emission signal passed a dichroic mirror (FT 395; Zeiss, Germany) and was filtered by a 510-nm bandpass filter (D510/40M; Chroma Technology). All fluorescence signals were recorded by a Quantem 512SC camera (Photometrics, USA; www.photometrics.com) using Visiview software (Visitron Systems). The Image J software package was used (FIJI version, National Institute of Health, https://imagej.net/Welcome), to correct images for background fluorescence, calculate FURA2 ratio values and obtain pseudocolored images. The FURA2-dextran signals are shown as the ratio of the emission signals obtained with excitation light of 345 nm and 390 nm.

### Data analysis

The membrane potential data recorded with the WinEDR, or the PULSE, software were imported and analyzed in Microsoft Excel. Average data and standard error of the mean (SE) are shown of a number of experiments, as given in the legends of the respective figures. All graphs were made with Igor pro software (www.wavemetrics.com).

## Results

### Nod-LCO- and Myc-LCO-induced membrane potential depolarizations

**Fig 1:**
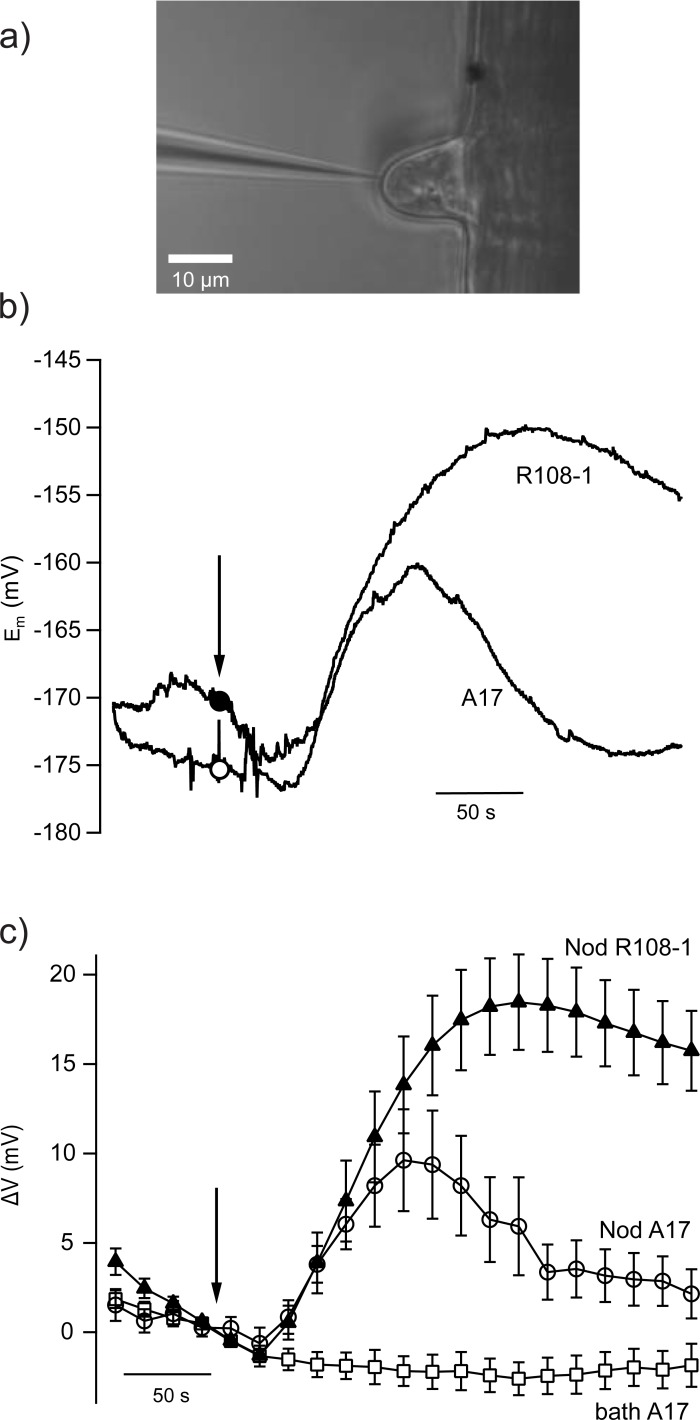
Nod-LCO induced plasma membrane potential depolarizations in root hairs of two *M*. *truncatula* genotypes. (A) Transmitted light image of a *M*. *tuncatula* root hair impaled with a microelectrode. Scale bar represents 10 μm. (B) Representative membrane potential changes evoked by application of 100 nM Nod-LCO (NodSM-IV, C16:2) to the bath solution. Membrane potential changes are shown of root hair cells of the genotypes A17 (closed circle) and R108-1 (open circle). (C) Average changes in plasma membrane potential of *M*. *truncatula* root hairs, stimulated with 100 nM Nod-LCOs (NodSM-IV, C16:2, S, triangles and open circles), or control solution (open rectangles). The time point of Nod-LCO application is indicated by the arrow. Data are shown for root hair cells of *M*. *truncatula* genotype A17 (n = 11, open circles) and R108-1 (n = 37, closed triangles). Error bars indicate SE.

The membrane potential depolarization of root hair cells represents one of the first steps evoked by Nod-LCOs in the host plants [28–30], but it is unknown if this response is provoked by Myc-LCOs. We tested the impact of a *Sinorhizobium meliloti* Nod-LCO (NodSM-IV, C16:2, S), on the membrane potential of *M*. *truncatula* roots using the micro electrode impalement technique (Fig 1A). Root hair cells were kept in bath solutions with Cs^+^ to block K^+^ uptake channels and hyperpolarize the plasma membrane potential. Under these conditions, membrane potentials were recorded that range from -138 mV to -187 mV (n = 37) in *M*. *truncatula* genotype R108-1, whereas slightly more hyperpolarized values from -156 mV to -224 mV were found in genotype A17 (n = 11). Stimulation with 100 nM of the Nod-LCO (NodSM-IV, C16:2, S) induced a transient depolarization in both genotypes, after a lag phase of 45 s (SE = 3.74, n = 32) in R108-1 and 34 s (SE = 2.6, n = 9) in A17 (Fig 1B and 1C). Despite of the less hyperpolarized membrane potential of R108-1, Nod-LCOs triggered a two-fold larger membrane depolarization in this genotype, as compared to A17 (Fig 1B and 1C). On average, the maximal depolarization was 21 mV (SE = 2.8, n = 37) in R108-1, whereas it was only 10 mV (SE = 2.7, n = 11) in A17. The time span to reach the depolarization maximum also varied, it was 211 s (SE = 10.7, n = 32) in R108-1, but only 109 s (SE = 7.9, n = 9) in A17.

**Fig 2:**
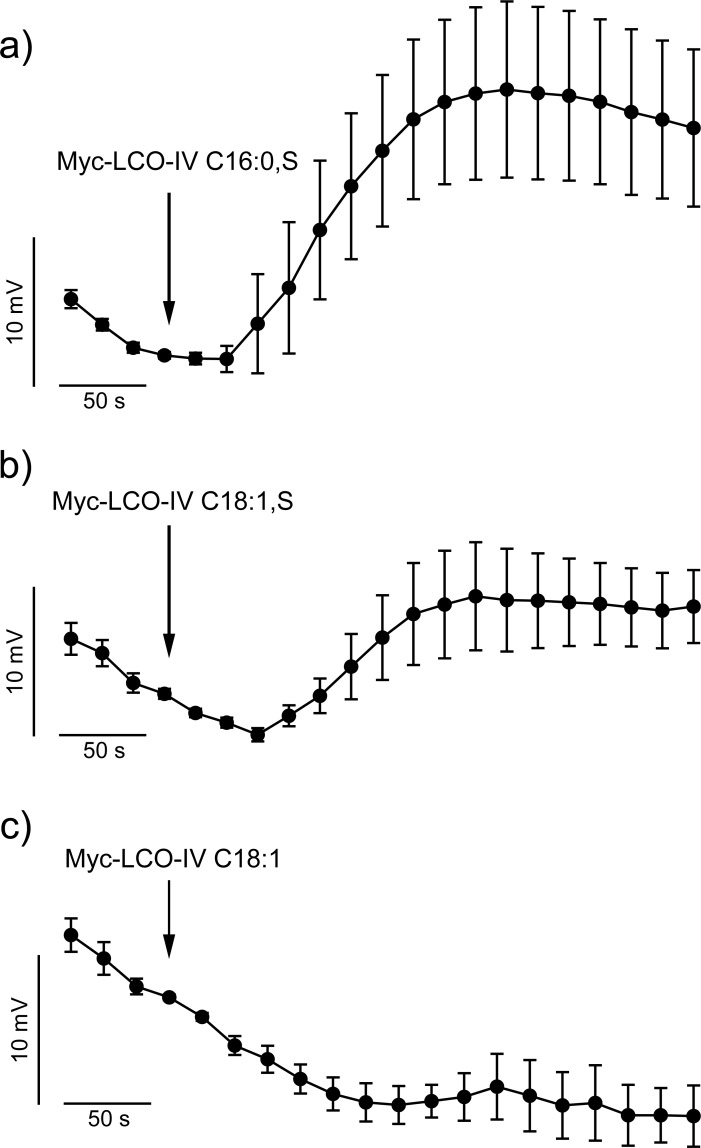
Impact of selected Myc-LCOs on the membrane potential of *M*. *truncatula*, genotype R108-1 root hair cells. (A-C) Average changes in membrane potential of *M*. *truncatula* genotype R108-1 root hairs, induced by the following three Myc-LCOs; (A) Myc-LCO-IV, C16:0, S (n = 9), (B) Myc-LCO-IV, C18:1, S (n = 8), (C) Myc-LCO-IV, C18:1 (n = 6). The traces represent average data ± SE. Note that the depolarization induced by LCO-IV C16:0, S is larger than that triggered by Myc-LCO-IV, C18:1, S and that 100 nM non-sulfated Myc-LCOs (LCO-IV, C18:1) had no effect on the membrane potential.

Myc-LCOs have a similar chemical nature as Nod-LCOs and to some extend both stimuli provoke the same responses in host plants [31]. Indeed, root hair cells of *M*. *truncatula* R108-1 depolarized in response to the sulfated Myc-LCO (Myc-LCO-IV, C16:0, S) with the same magnitude, as to the Nod-LCO (NodSM-IV, C16:2, S; 19 mV, SE = 6.1, n = 9) (Fig 2A). However, the response to another Myc-LCO (Myc-LCO-IV, C18:1, S) was smaller (9 mV, SE = 3.6, n = 8) (Fig 2B). In contrast to both sulfated Myc-LCOs, the non-sulfated Myc-LCO (Myc-LCO-IV, C18:1) did not affect the membrane potential of *M*. *truncatula* root hair cells (Fig 2C).

**Fig 3:**
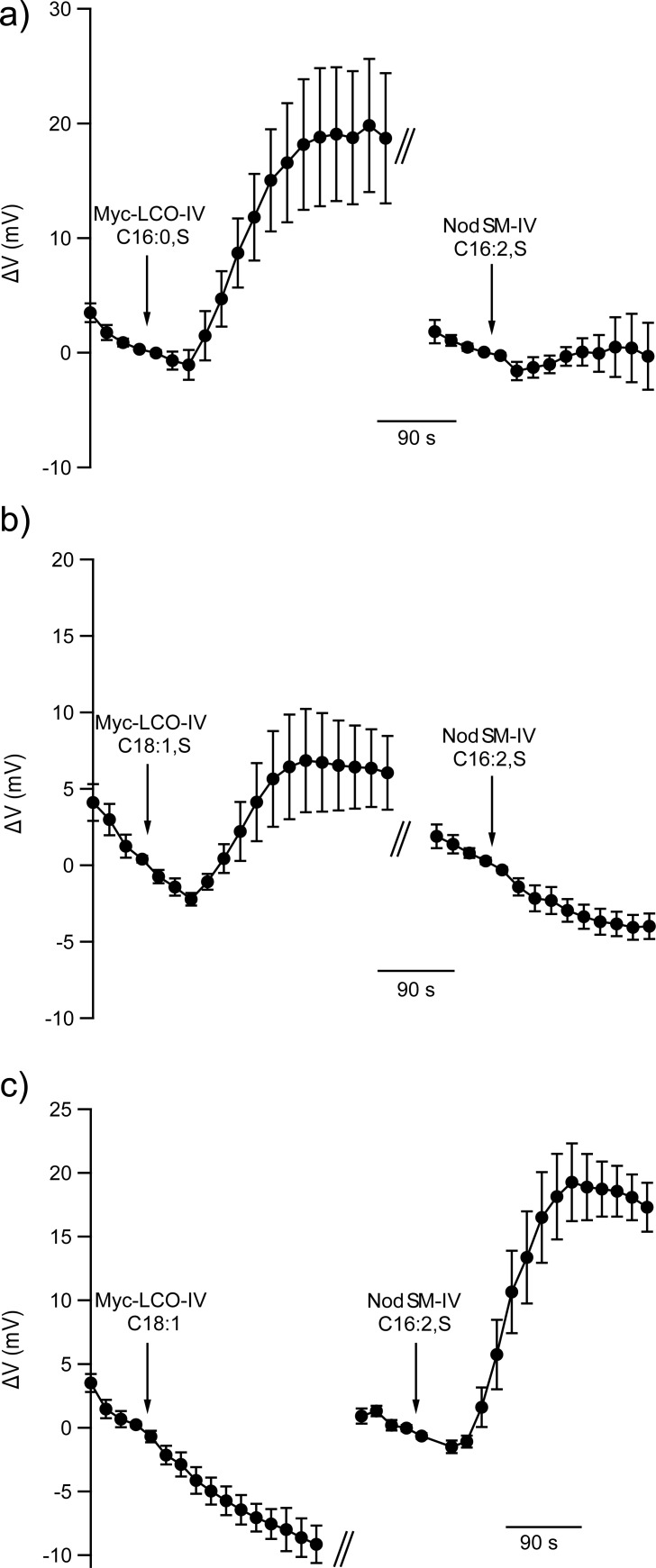
Interference of Myc-LCOs with Nod-LCO-induced depolarization of root hairs. **(A-C)** Average changes in membrane potential of *M*. *truncatula* root hairs, first stimulated with Myc-LCOs; **(A)** Myc-LCO-IV C16:0, S, (n = 7) and **(B)** Myc-LCO-IV C18:1, S (n = 8), **(C)** Myc-LCO-IV C18:1, (n = 7), followed by application of the Nod-LCO (NodSM-IV, C16:2, S). Error bars represent SE.

Because of their similarities in chemical nature, Nod- and Myc-LCOs may compete for a single perception mechanism in root hairs. This possibility was tested by application of a sulfated Myc-LCO, prior to stimulation with Nod-LCOs. In line with a common perception module, the presence of the sulfated Myc-LCO repressed plasma membrane responses to Nod-LCOs (Fig 3A and 3B). However, non-sulfated Myc-LCOs were not effective, as root hairs remained responsive to the Nod-LCOs, despite of the presence of the non-sulfated Myc-LCOs (Fig 3C).

### Nod-LCO-induced membrane depolarization precedes cytosolic calcium-spiking

**Fig 4:**
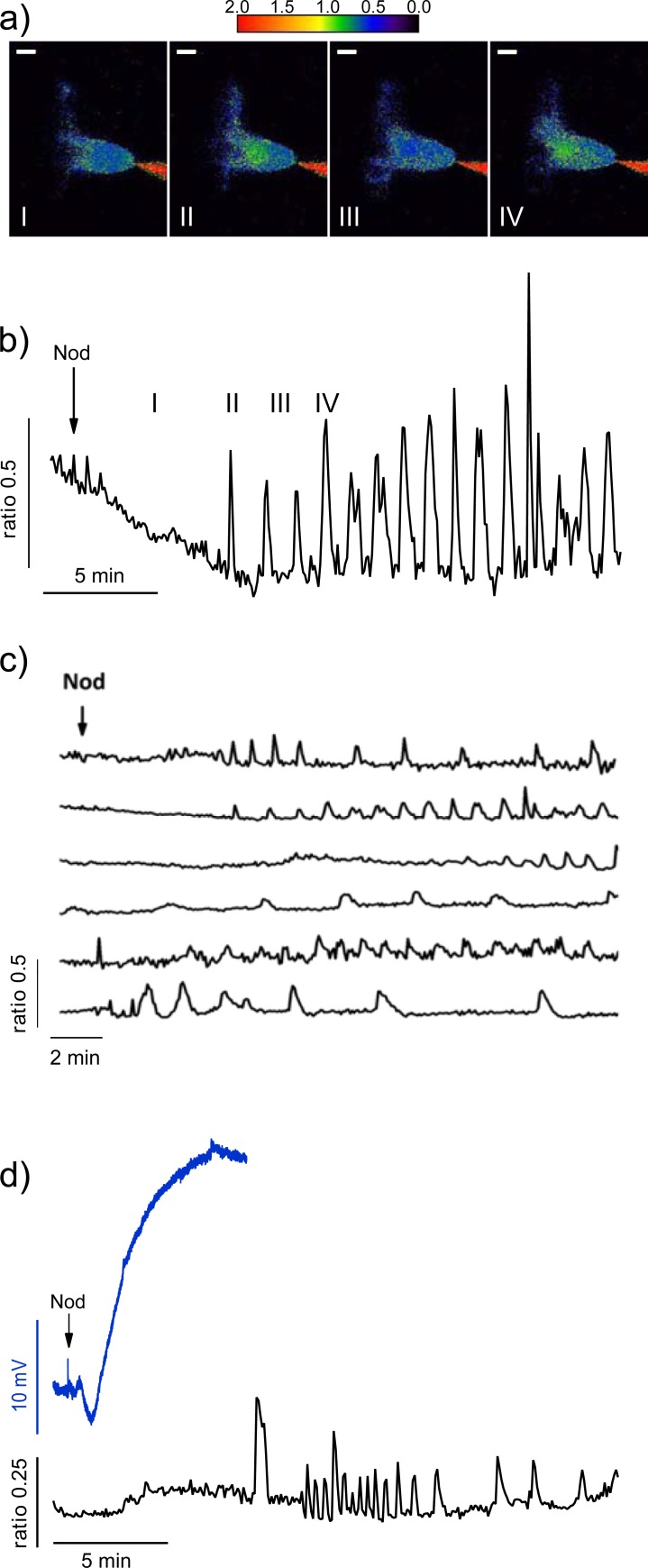
The Nod-LCO-induced depolarization is not linked to cytosolic Ca^2+^ signals. **(A)** Pseudocolour images of a *M*. *truncatula* root hair, which was current injected with FURA2-Dextran and stimulated with Nod-LCO (NodSM-IV, C16:2, S). The FURA2 F345/390 excitation wavelength ratio is color coded as indicated by the calibration bar on the right. Images were obtained several minutes after the application of Nod-LCO, as shown in **B** (symbols in A and B correspond). The scale bars in A represent 10 μm. (**B**) Changes in the FURA2 F345/390 ratio plotted against time, for the same root hair cell as shown in **A**, symbols correspond. **(C)** Changes in FURA2 F345/390 fluorescence ratio, triggered by Nod-LCO application, as indicated by the arrow, plotted against time, for 6 root hair cells. Note, that the Nod-LCO evokes repetitive spikes in the cytosolic Ca^2+^-concentration (based on rises in the FURA2 F345/390 ratio), which start approximately 4 min. after Nod-LCO application and proceed with variable magnitude and time intervals. **(D)** Simultaneous recording of the plasma membrane potential (upper trace) and FURA2 F345/390 ratio in a *M*. *truncatula* root hair cell. Note that application of the Nod-LCO (indicated by the arrow) causes a membrane potential depolarization that occurs much earlier as the first peak in the cytosolic free Ca^2+^ concentration (based on the rise of the FURA2 F345/390 ratio).

Stimulus-induced depolarizations of the plant plasma membrane are often linked to a transient elevation of the cytosolic Ca^2+^ concentration [32, 33]. The ratiometric Ca^2+^ indicator FURA2 is rapidly transported into vacuoles of root hairs of *M*. *truncatula* and we therefore current injected FURA2-dextran with micro electrodes, which results in a stable cytoplasmic localization of the dye (Fig 4A). Stimulation with 100 nM Nod-LCO evoked regular oscillations of the cytosolic free Ca^2+^concentration after a lag phase of approximately 8 min (Fig 4B and 4C). Pseudocolour images, indicating the FURA2 345 nm/390 nm excitation ratio, show that the repetitive increases in cytosolic Ca^2+^ concentration occurred predominantly in a subcellular compartment that includes the nucleus (Fig 4A). Repetitive spikes of the intracellular Ca^2+^ concentration were observed in 16 out of 23 cells. The frequency and the shape of the Ca^2+^ peaks varied between cells, but no elevation of the cytosolic Ca^2+^ concentration was observed within the first minutes, during which the plasma membrane depolarizes (compare Figs 1 and 4). This indicates that the Nod-LCO-induced depolarization of the plasma membrane precedes the repetitive transient Ca^2+^ elevations in the cytosol of root hairs.

We tested the hypothesis that the Nod-LCO-evoked depolarization occurs before the Ca^2+^ signals are provoked, by re-impaling root hairs that were loaded with FURA2-dextran. In these cells, a KCl-filled electrode measures the membrane potential, while the cytosolic Ca^2+^ level was monitored with FURA2 (Fig 4D). This approach revealed that Nod-LCOs first caused a depolarization of the plasma membrane, which is followed by the occurrence of repetitive Ca^2+^ spikes that are associated with the nucleus.

## Discussion

### Membrane depolarization

A transient depolarization of the plasma membrane is an early response evoked by several biotic stimuli, in a variety of cell types. Previously, these responses have been described for Nod-LCOs, as well as the structurally related chitin fragments and several other MAMPs [28–30, 33–37]. Our study revealed that sulfated Myc-LCOs trigger a transient depolarization of *M*. *truncatula* root hairs, but non-sulfated Myc-LCOs did not. At a concentration of 100 nM, non-sulfated Myc-LCOs can stimulate root branching (RBS) [9], as well as the expression of symbiosis specific genes [22] and provoke Ca^2+^ signals in cells of lateral roots [38], but they do not evoke a plasma membrane depolarization (Fig 2C). The depolarization of root cells may thus depend on another perception mechanism as the one that provokes Ca^2+^ signals, root branching and gene expression.

In contrast to the recognition of non-sulfated Myc-LCOs, the perception mechanism of sulfated Myc-LCOs is likely to be shared with that of Nod-LCOs, as the successive application of two stimuli resulted only in a single transient depolarization (Fig 3A and 3B). This suggests that both types of stimuli are acting through the same signaling pathway. A similar conclusion was drawn by Sun et al. (2015), who measured similar cytosolic Ca^2+^ signals evoked by Myc- or Nod-LCOs [38].

### Cytosolic Ca^2+^-signals

Changes in the cytosolic Ca^2+^ concentration that occur simultaneously with the plasma membrane depolarization have been recorded earlier with intracellular ion-selective electrodes [39]. Depending on the side of impalement, these electrodes either recorded an increase, or decrease, of the cytosolic Ca^2+^ level in growing root hair cells. These early Ca^2+^ signals were not detected by other researchers that used FURA2-dextran in *M*. *truncatula*, suggesting that they are confined to the plasma membrane [40–42]. Instead, FURA2 or genetically encoded Ca^2+^-sensors detected repetitive elevations of the Ca^2+^ level after stimulation with Nod-LCOs, which occur at a later stage as the plasma membrane depolarization [27, 40–44].

Here we show that in root hairs of *M*. *truncatula*, the first transient increase of the cytosolic Ca^2+^ concentration was not observed within 4 min. after application of Nod-LCOs (Fig 4B). Nod-LCO-induced depolarizations occurred earlier and normally peaked 3 min after application of the stimulus (Fig 1B). We thus conclude that the depolarization precedes the cytosolic Ca^2+^ signals associated with the nucleus, which was confirmed by simultaneous measurement of both responses (Fig 4D). The repetitive Ca^2+^ peaks are thus unlikely to provoke membrane potential changes, but instead activate Ca^2+^/calmodulin-dependent protein kinases (CCaMKs) that are required for nodule formation [45–48]. Myc-LCOs trigger similar Ca^2+^ signals in root hair cells [38], which are also decoded by CCaMK leading to arbuscular branching [49].

### Outlook

A plasma membrane depolarization is the earliest response that can be observed upon stimulation of *M*. *truncatula* root cells with Nod- or Myc-LCOs. This response even precedes the cytosolic Ca^2+^ signals in root hairs, which are a central element in the signaling chain that leads to nodule formation [31]. Even though the Nod-LCO-induced depolarization already was recognized in 1992 [28], we still do not know the nature of the ion channels that are addressed by the signaling pathway. Felle et al. (1998) showed that during the depolarization anions are released from roots and thus suggested that anion channels are activated by Nod-LCOs [50]. In *Arabidopsis* the SLAC/SLAH- and ALMT-gene families encode anion channels, of which all SLAC/SLAH- and several ALMT-members are targetted to the plasma membrane. Target repression of homologous genes in *M*. *truncatula* may reveal if genes of these famlies indeed encode anion channels that are activated by Nod- and Myc-LCOs.

The magnitude of the Nod-LCO-induced depolaization is strongly dependent on the genotype of the *M*. *truncatula* line that is studied (Fig 1B). Genotypic variation thus may be exploited to search for genes that are involved in the Nod- and Myc-LCO-dependent membrane potential change. This experimental approach may take advantage of mutant collections that have been generated for *M*. *truncatula* with use of the Tnt1 retrotransposon [51]. Future studies that adress the variation in plasma membrane responses, thus are likely to give insights in the sequence of events that occurs within the first minutes after stimulation with LCOs extruded by symbiotic bacteria and fungi.

## Supporting information

S1 Table Data of Nod-LCO induced plasma membrane potential depolarizations in root hairs of two *M. truncatula* genotypes, as shown in Fig 1. (XLSX)

S2 Table Data that reveal the impact of selected Myc-LCOs on the membrane potential of *M. truncatula*, genotype R108-1 root hair cells, as shown in Fig 2. (XLSX)

S3 Table Data on the interference of Myc-LCOs, with Nod-LCO-induced depolarization of root hairs, as shown in Fig 3. (XLSX)

S4 Table Data that reveal that the Nod-LCO-induced depolarization is not linked to cytosolic Ca^2+^ signals, as shown in Fig 4. (XLSX)

## References

[1] Remy W, Taylor TN, Hass H, Kerp H. Four hundred-million-year-old vesicular arbuscular mycorrhizae. Proceedings of the National Academy of Sciences of the United States of America. 1994;91(25):11841–3. 11607500 10.1073/pnas.91.25.11841 PMC45331

[2] Smith S, Read D. Mycorrhizal Symbiosis. Academic Press, London, UK 2008;3rd ed.

[3] Trinick MJ. Structure of nitrogen-fixing nodules formed by Rhizobium on roots of *Parasponia andersonii* Planch. Canadian journal of microbiology. 1979;25(5):565–78. 476539 10.1139/m79-082

[4] Op den Camp R, Streng A, De Mita S, Cao Q, Polone E, Liu W, et al LysM-type mycorrhizal receptor recruited for rhizobium symbiosis in nonlegume Parasponia. Science. 2011;331(6019):909–12. doi: 10.1126/science.1198181 21205637 10.1126/science.1198181

[5] Harrison MJ. Signaling in the arbuscular mycorrhizal symbiosis. Annual Review of Microbiology. 2005;59:19–42. doi: 10.1146/annurev.micro.58.030603.123749 16153162 10.1146/annurev.micro.58.030603.123749

[6] Oldroyd GE, Downie JA. Coordinating nodule morphogenesis with rhizobial infection in legumes. Annual review of plant biology. 2008;59:519–46. doi: 10.1146/annurev.arplant.59.032607.092839 18444906 10.1146/annurev.arplant.59.032607.092839

[7] Mus F, Crook MB, Garcia K, Garcia Costas A, Geddes BA, Kouri ED, et al Symbiotic nitrogen fixation and the challenges to its extension to nonlegumes. Applied and environmental microbiology. 2016;82(13):3698–710. doi: 10.1128/AEM.01055-16 27084023 10.1128/AEM.01055-16 PMC4907175

[8] Lerouge P, Roche P, Faucher C, Maillet F, Truchet G, Prome JC, et al Symbiotic host-specificity of *Rhizobium meliloti* is determined by a sulphated and acylated glucosamine oligosaccharide signal. Nature. 1990;344(6268):781–4. doi: 10.1038/344781a0 2330031 10.1038/344781a0

[9] Maillet F, Poinsot V, Andre O, Puech-Pages V, Haouy A, Gueunier M, et al Fungal lipochitooligosaccharide symbiotic signals in arbuscular mycorrhiza. Nature. 2011;469(7328):58–63. doi: 10.1038/nature09622 21209659 10.1038/nature09622

[10] Roche P, Debelle F, Maillet F, Lerouge P, Faucher C, Truchet G, et al Molecular basis of symbiotic host specificity in *Rhizobium meliloti*: nodH and nodPQ genes encode the sulfation of lipo-oligosaccharide signals. Cell. 1991;67(6):1131–43. 1760841 10.1016/0092-8674(91)90290-f

[11] Denarie J, Debelle F, Prome JC. Rhizobium lipo-chitooligosaccharide nodulation factors: signaling molecules mediating recognition and morphogenesis. Annual Review of Biochemistry. 1996;65:503–35. doi: 10.1146/annurev.bi.65.070196.002443 8811188 10.1146/annurev.bi.65.070196.002443

[12] Miller JB, Oldroyd GED. The role of diffusible signals in the establishment of rhizobial and mycorrhizal symbioses. Signal Commun Plants 2012;10:1–30.

[13] Limpens E, Franken C, Smit P, Willemse J, Bisseling T, Geurts R. LysM domain receptor kinases regulating rhizobial Nod factor-induced infection. Science. 2003;302(5645):630–3. doi: 10.1126/science.1090074 12947035 10.1126/science.1090074

[14] Arrighi JF, Barre A, Ben Amor B, Bersoult A, Soriano LC, Mirabella R, et al The *Medicago truncatula* lysin motif-receptor-like kinase gene family includes NFP and new nodule-expressed genes. Plant Physiology. 2006;142(1):265–79. doi: 10.1104/pp.106.084657 16844829 10.1104/pp.106.084657 PMC1557615

[15] Madsen EB, Madsen LH, Radutoiu S, Olbryt M, Rakwalska M, Szczyglowski K, et al A receptor kinase gene of the LysM type is involved in legume perception of rhizobial signals. Nature. 2003;425(6958):637–40. doi: 10.1038/nature02045 14534591 10.1038/nature02045

[16] Radutoiu S, Madsen LH, Madsen EB, Felle HH, Umehara Y, Gronlund M, et al Plant recognition of symbiotic bacteria requires two LysM receptor-like kinases. Nature. 2003;425(6958):585–92. doi: 10.1038/nature02039 14534578 10.1038/nature02039

[17] Endre G, Kereszt A, Kevei Z, Mihacea S, Kalo P, Kiss GB. A receptor kinase gene regulating symbiotic nodule development. Nature. 2002;417(6892):962–6. doi: 10.1038/nature00842 12087406 10.1038/nature00842

[18] Stracke S, Kistner C, Yoshida S, Mulder L, Sato S, Kaneko T, et al A plant receptor-like kinase required for both bacterial and fungal symbiosis. Nature. 2002;417(6892):959–62. doi: 10.1038/nature00841 12087405 10.1038/nature00841

[19] Miya A, Albert P, Shinya T, Desaki Y, Ichimura K, Shirasu K, et al CERK1, a LysM receptor kinase, is essential for chitin elicitor signaling in Arabidopsis. Proceedings of the National Academy of Sciences of the United States of America. 2007;104(49):19613–8. doi: 10.1073/pnas.0705147104 18042724 10.1073/pnas.0705147104 PMC2148337

[20] Boller T, Felix G. A renaissance of elicitors: perception of microbe-associated molecular patterns and danger signals by pattern-recognition receptors. Annual Review of Plant Biology. 2009;60:379–406. doi: 10.1146/annurev.arplant.57.032905.105346 19400727 10.1146/annurev.arplant.57.032905.105346

[21] Trieu AT, Burleigh SH, Kardailsky IV, Maldonado-Mendoza IE, Versaw WK, Blaylock LA, et al Transformation of *Medicago truncatula* via infiltration of seedlings or flowering plants with Agrobacterium. The Plant journal. 2000;22(6):531–41. . 10886773 10.1046/j.1365-313x.2000.00757.x

[22] Czaja LF, Hogekamp C, Lamm P, Maillet F, Martinez EA, Samain E, et al Transcriptional responses toward diffusible signals from symbiotic microbes reveal MtNFP- and MtDMI3-dependent reprogramming of host gene expression by arbuscular mycorrhizal fungal lipochitooligosaccharides. Plant Physiology. 2012;159(4):1671–85. doi: 10.1104/pp.112.195990 22652128 10.1104/pp.112.195990 PMC3425205

[23] Ohsten Rasmussen M, Hogg B, Bono JJ, Samain E, Driguez H. New access to lipo-chitooligosaccharide nodulation factors. Organic & Biomolecular Chemistry. 2004;2(13):1908–10. doi: 10.1039/b403575e 15227544 10.1039/b403575e

[24] Dempster J. Computer Analysis of Electrophysiological Signals. Academic Press, London, UK 1993.

[25] Dempster J. The Laboratory Computer: A practical guide for neuroscientists and physiologists. Academic Press, London, UK 2001.

[26] Levchenko V, Guinot DR, Klein M, Roelfsema MRG, Hedrich R, Dietrich P. Stringent control of cytoplasmic Ca^2+^ in guard cells of intact plants compared to their counterparts in epidermal strips or guard cell protoplasts. Protoplasma. 2008;233(1–2):61–72. doi: 10.1007/s00709-008-0307-x 18648729 10.1007/s00709-008-0307-x

[27] Ehrhardt DW, Wais R, Long SR. Calcium spiking in plant root hairs responding to Rhizobium nodulation signals. Cell. 1996;85(5):673–81. 8646776 10.1016/s0092-8674(00)81234-9

[28] Ehrhardt DW, Atkinson EM, Long SR. Depolarization of alfalfa root hair membrane potential by *Rhizobium meliloti* Nod factors. Science. 1992;256(5059):998–1000. 10744524 10.1126/science.10744524

[29] Kurkdjian AC. Role of the differentiation of root epidermal cells in Nod factor (from *Rhizobium meliloti*)-induced root-hair depolarization of *Medicago sativa*. Plant physiology. 1995;107(3):783–90. 12228403 10.1104/pp.107.3.783 PMC157194

[30] Felle HH, Kondorosi E, Kondorosi A, Schultze M. Nod signal-induced plasma membrane potential changes in alfalfa root hairs are differentially sensitive to structural modifications of the lipochitooligosaccharide. The Plant Journal. 1995;7(6):939–47.

[31] Oldroyd GE. Speak, friend, and enter: signalling systems that promote beneficial symbiotic associations in plants. Nature Reviews Microbiology. 2013;11(4):252–63. doi: 10.1038/nrmicro2990 23493145 10.1038/nrmicro2990

[32] Kudla J, Batistic O, Hashimoto K. Calcium signals: The lead currency of plant information processing. Plant Cell. 2010;22(3):541–63. doi: 10.1105/tpc.109.072686 20354197 10.1105/tpc.109.072686 PMC2861448

[33] Roelfsema MR, Hedrich R, Geiger D. Anion channels: master switches of stress responses. Trends in plant science. 2012;17(4):221–9. doi: 10.1016/j.tplants.2012.01.009 22381565 10.1016/j.tplants.2012.01.009

[34] Wendehenne D, Lamotte O, Frachisse JM, Barbier-Brygoo H, Pugin A. Nitrate efflux is an essential component of the cryptogein signaling pathway leading to defense responses and hypersensitive cell death in tobacco. Plant Cell. 2002;14(8):1937–51. doi: 10.1105/tpc.002295 12172032 10.1105/tpc.002295 PMC151475

[35] Jeworutzki E, Roelfsema MR, Anschutz U, Krol E, Elzenga JT, Felix G, et al Early signaling through the Arabidopsis pattern recognition receptors FLS2 and EFR involves Ca^2+^-associated opening of plasma membrane anion channels. The Plant journal: for cell and molecular biology. 2010;62(3):367–78. doi: 10.1111/j.1365-313X.2010.04155.x 20113440 10.1111/j.1365-313X.2010.04155.x

[36] Koers S, Guzel-Deger A, Marten I, Roelfsema MR. Barley mildew and its elicitor chitosan promote closed stomata by stimulating guard-cell S-type anion channels. The Plant journal. 2011;68(4):670–80. doi: 10.1111/j.1365-313X.2011.04719.x 21781196 10.1111/j.1365-313X.2011.04719.x

[37] Ye W, Muroyama D, Munemasa S, Nakamura Y, Mori IC, Murata Y. Calcium-dependent protein kinase CPK6 positively functions in induction by yeast elicitor of stomatal closure and inhibition by yeast elicitor of light-induced stomatal opening in Arabidopsis. Plant Physiology. 2013;163(2):591–9. doi: 10.1104/pp.113.224055 23922271 10.1104/pp.113.224055 PMC3793040

[38] Sun J, Miller JB, Granqvist E, Wiley-Kalil A, Gobbato E, Maillet F, et al Activation of symbiosis signaling by arbuscular mycorrhizal fungi in legumes and rice. The Plant cell. 2015;27(3):823–38. doi: 10.1105/tpc.114.131326 25724637 10.1105/tpc.114.131326 PMC4558648

[39] Felle HH, Kondorosi E, Kondorosi A, Schultze M. Nod factors modulate the concentration of cytosolic free calcium differently in growing and non-growing root hairs of *Medicago sativa* L. Planta. 1999;209(2):207–12. doi: 10.1007/s004250050624 . 10436223 10.1007/s004250050624

[40] Wais RJ, Galera C, Oldroyd G, Catoira R, Penmetsa RV, Cook D, et al Genetic analysis of calcium spiking responses in nodulation mutants of Medicago truncatula. Proceedings of the National Academy of Sciences of the United States of America. 2000;97(24):13407–12. doi: 10.1073/pnas.230439797 11078514 10.1073/pnas.230439797 PMC27237

[41] Wais RJ, Keating DH, Long SR. Structure-function analysis of nod factor-induced root hair calcium spiking in Rhizobium-legume symbiosis. Plant physiology. 2002;129(1):211–24. doi: 10.1104/pp.010690 12011352 10.1104/pp.010690 PMC155885

[42] Capoen W, Den Herder J, Sun J, Verplancke C, De Keyser A, De Rycke R, et al Calcium spiking patterns and the role of the calcium/calmodulin-dependent kinase CCaMK in lateral root base nodulation of Sesbania rostrata. The Plant cell. 2009;21(5):1526–40. doi: 10.1105/tpc.109.066233 19470588 10.1105/tpc.109.066233 PMC2700542

[43] Miwa H, Sun J, Oldroyd GE, Downie JA. Analysis of calcium spiking using a cameleon calcium sensor reveals that nodulation gene expression is regulated by calcium spike number and the developmental status of the cell. The Plant journal. 2006;48(6):883–94. doi: 10.1111/j.1365-313X.2006.02926.x 17227545 10.1111/j.1365-313X.2006.02926.x

[44] Morieri G, Martinez EA, Jarynowski A, Driguez H, Morris R, Oldroyd GE, et al Host-specific Nod-factors associated with Medicago truncatula nodule infection differentially induce calcium influx and calcium spiking in root hairs. New Phytologist. 2013;200(3):656–62. doi: 10.1111/nph.12475 24015832 10.1111/nph.12475 PMC3908372

[45] Levy J, Bres C, Geurts R, Chalhoub B, Kulikova O, Duc G, et al A putative Ca^2+^ and calmodulin-dependent protein kinase required for bacterial and fungal symbioses. Science. 2004;303(5662):1361–4. doi: 10.1126/science.1093038 14963335 10.1126/science.1093038

[46] Mitra RM, Gleason CA, Edwards A, Hadfield J, Downie JA, Oldroyd GE, et al A Ca^2+^/calmodulin-dependent protein kinase required for symbiotic nodule development: Gene identification by transcript-based cloning. Proceedings of the National Academy of Sciences of the United States of America. 2004;101(13):4701–5. doi: 10.1073/pnas.0400595101 15070781 10.1073/pnas.0400595101 PMC384810

[47] Gleason C, Chaudhuri S, Yang T, Munoz A, Poovaiah BW, Oldroyd GE. Nodulation independent of rhizobia induced by a calcium-activated kinase lacking autoinhibition. Nature. 2006;441(7097):1149–52. doi: 10.1038/nature04812 16810256 10.1038/nature04812

[48] Tirichine L, Imaizumi-Anraku H, Yoshida S, Murakami Y, Madsen LH, Miwa H, et al Deregulation of a Ca^2+^/calmodulin-dependent kinase leads to spontaneous nodule development. Nature. 2006;441(7097):1153–6. doi: 10.1038/nature04862 16810257 10.1038/nature04862

[49] Pimprikar P, Carbonnel S, Paries M, Katzer K, Klingl V, Bohmer MJ, et al A CCaMK-CYCLOPS-DELLA complex activates transcription of RAM1 to regulate arbuscule branching. Current Biology. 2016;26(8):1126 doi: 10.1016/j.cub.2016.04.021 27115681 10.1016/j.cub.2016.04.021

[50] Felle HH, Kondorosi E, Kondorosi A, Schultze M. The role of ion fluxes in Nod factor signalling in *Medicago sativa*. The Plant Journal. 1998;13(4):455–63.

[51] Tadege M, Wen J, He J, Tu H, Kwak Y, Eschstruth A, et al Large-scale insertional mutagenesis using the Tnt1 retrotransposon in the model legume *Medicago truncatula*. The Plant journal. 2008;54(2):335–47. doi: 10.1111/j.1365-313X.2008.03418.x 18208518 10.1111/j.1365-313X.2008.03418.x

